# Replicative and non-replicative mechanisms in the formation of clustered CNVs are indicated by whole genome characterization

**DOI:** 10.1371/journal.pgen.1007780

**Published:** 2018-11-12

**Authors:** Lusine Nazaryan-Petersen, Jesper Eisfeldt, Maria Pettersson, Johanna Lundin, Daniel Nilsson, Josephine Wincent, Agne Lieden, Lovisa Lovmar, Jesper Ottosson, Jelena Gacic, Outi Mäkitie, Ann Nordgren, Francesco Vezzi, Valtteri Wirta, Max Käller, Tina Duelund Hjortshøj, Cathrine Jespersgaard, Rayan Houssari, Laura Pignata, Mads Bak, Niels Tommerup, Elisabeth Syk Lundberg, Zeynep Tümer, Anna Lindstrand

**Affiliations:** 1 Wilhelm Johannsen Center for Functional Genome Research, Institute of Cellular and Molecular Medicine, University of Copenhagen, Copenhagen, Denmark; 2 Department of Molecular Medicine and Surgery, Center for Molecular Medicine, Karolinska Institute, Stockholm, Sweden; 3 Science for Life Laboratory, Karolinska Institutet Science Park, Solna, Sweden; 4 Department of Clinical Genetics, Karolinska University Hospital, Stockholm, Sweden; 5 Department of Clinical Genetics, Sahlgrenska University Hospital, Gothenburg, Sweden; 6 Department of Clinical Genetics, Linköping University Hospital, Linköping, Sweden; 7 Children’s Hospital, University of Helsinki and Helsinki University Hospital, Helsinki, Finland; 8 Folkhälsan Institute of Genetics, Helsinki, Finland; 9 SciLifeLab, Department of Biochemistry and Biophysics, Stockholm University, Stockholm, Sweden; 10 SciLifeLab, School of Engineering Sciences in Chemistry, Biotechnology and Health, KTH Royal Institute of Technology, Stockholm, Sweden; 11 SciLifeLab, Department of Microbiology, Tumor and Cell biology, Karolinska Institutet, Stockholm, Sweden; 12 Kennedy Center, Department of Clinical Genetics, Copenhagen University Hospital, Rigshospitalet, Glostrup, Denmark; 13 Department of Clinical Medicine, Faculty of Health and Medical Sciences, University of Copenhagen, Denmark; University of Pennsylvania, UNITED STATES

## Abstract

Clustered copy number variants (CNVs) as detected by chromosomal microarray analysis (CMA) are often reported as germline chromothripsis. However, such cases might need further investigations by massive parallel whole genome sequencing (WGS) in order to accurately define the underlying complex rearrangement, predict the occurrence mechanisms and identify additional complexities. Here, we utilized WGS to delineate the rearrangement structure of 21 clustered CNV carriers first investigated by CMA and identified a total of 83 breakpoint junctions (BPJs). The rearrangements were further sub-classified depending on the patterns observed: I) Cases with only deletions (n = 8) often had additional structural rearrangements, such as insertions and inversions typical to chromothripsis; II) cases with only duplications (n = 7) or III) combinations of deletions and duplications (n = 6) demonstrated mostly interspersed duplications and BPJs enriched with microhomology. In two cases the rearrangement mutational signatures indicated both a breakage-fusion-bridge cycle process and haltered formation of a ring chromosome. Finally, we observed two cases with *Alu*- and LINE-mediated rearrangements as well as two unrelated individuals with seemingly identical clustered CNVs on 2p25.3, possibly a rare European founder rearrangement.

In conclusion, through detailed characterization of the derivative chromosomes we show that multiple mechanisms are likely involved in the formation of clustered CNVs and add further evidence for chromoanagenesis mechanisms in both “simple” and highly complex chromosomal rearrangements. Finally, WGS characterization adds positional information, important for a correct clinical interpretation and deciphering mechanisms involved in the formation of these rearrangements.

## Author summary

Clustered copy number variants (CNVs) as detected by chromosomal microarray are often reported as germline chromoanagenesis. However, such cases might need further investigation by whole genome sequencing (WGS) to accurately resolve the complexity of the structural rearrangement and predict underlying mutational mechanisms. Here, we used WGS to characterize 83 breakpoint-junctions (BPJs) from 21 clustered CNVs, and outlined the rearrangement connectivity pictures. Cases with only deletions often had additional structural rearrangements, such as insertions and inversions, which could be a result of multiple double-strand DNA breaks followed by non-homologous repair, typical to chromothripsis. In contrast, cases with only duplications or combinations of deletions and duplications, demonstrated mostly interspersed duplications and BPJs enriched with microhomology, consistent with serial template switching during DNA replication (chromoanasynthesis). Only two rearrangements were repeat mediated. In aggregate, our results suggest that multiple CNVs clustered on a single chromosome may arise through either chromothripsis or chromoanasynthesis.

## Introduction

Structural variants (SVs) contribute to genomic diversity in human [1] and include copy number variants (CNVs) (deletions, duplications), as well as copy number neutral (balanced) variants (inversions and translocations), and more complex rearrangements, resulting from chromothripsis and/or chromoanasynthesis [2,3]. Complex SVs (complex chromosomal rearrangements, CCRs) often result in congenital and developmental abnormalities, as well as in cancer development, although carriers with unaffected phenotypes have also been reported [4].

A rare phenomenon regularly observed in clinical genetic diagnostic laboratories is multiple CNVs co-localizing on the same chromosome. Even though a chromosomal microarray (CMA) may identify such rearrangements, further characterization with whole genome sequencing (WGS) may be useful. A previous WGS study of two closely located duplications revealed additional copy-neutral complex genomic rearrangements associated with paired-duplications, such as inverted fragments, duplications with a nested deletion and other complexities, which were cryptic to CMA [5].

Proposed mechanisms that could explain the formation of multiple CNVs on the same chromosome include chromothripsis and chromoanasynthesis [6,7] while the term chromoanagenesis, a form of chromosome rebirth, describe the two phenomena independent of the underlying mechanism [8].

Chromothripsis is a chromosome shattering phenomenon, where part of or an entire chromosome, or few chromosomes, are fragmented into multiple pieces and reassembled in a random order and orientation resulting in complex genomic rearrangements [9]. During this process, some of the generated fragments can be lost resulting in heterozygous deletions. One of the distinctive features of chromothripsis is that the rearrangement breakpoints (BPs) are localized to relatively small genomic regions, usually spanning a few Mb. The causes of such clustered fragmentations are still unclear, however some studies suggested that chromothripsis could be generated through the physical isolation of chromosomes within micronuclei, where the “trapped” lagging chromosome(s) undergo defective DNA replication and repair, resulting in chromosome pulverization [10,11]. Others hypothesized that the clustered DNA double-strand breaks (DSBs) during chromothripsis could be initiated by ionizing radiation [9,12], breakage-fusion-bridge cycle associated with telomere attrition [9,13], aborted apoptosis [14], as well as endogenous endonucleases [15]. The highly characteristic breakpoint-junction (BPJ) sequences in the derivative chromosomes point to non-homologous end-joining (NHEJ) [16] or microhomology-mediated end-joining (MMEJ) [17] as being likely underlying repair mechanisms for rejoining of the shattered DNA fragments [9,18,19]. Although non-allelic homologous recombination (NAHR) was excluded as a chromothripsis repair mechanism [20], our recent report showed that homologous *Alu* elements may also mediate germline chromothripsis [15]. Chromothripsis was deciphered by the help of whole genome next generation sequencing technologies (WGS) in microscopic complex chromosomal rearrangements involving three or more BPs [18,19,21,22], as well as in microscopically balanced reciprocal translocations [23,24].

Chromoanasynthesis [25], was described by high resolution chromosome microarray analysis (CMA) and refers to clustered copy number changes, including deletions, duplications, and triplications, that are flanked by regions of normal dosage state. Small templated insertions and microhomologies found at most BPJs pinpointed that chromoanasynthesis likely involves replication failures, such as fork stalling and template switching (FoSTeS) [26] and/or microhomology-mediated break-induced replication (MMBIR) [27]. Another rare but distinct underlying mechanism of formation is atypical chromoanasynthesis that seems to only involve single chromosomes and exclusively generate duplications [28], either clustering on one chromosome arm or scattered throughout the entire chromosome.

It has also been shown that clustered duplications confined to a single chromosome may not only be integrated into the chromosome-of-origin in tandem, but could be integrated at multiple positions in the derivative chromosome and have non-templated insertions at the BPJs, indicating a different mutational mechanism, such as alternative NHEJ mediated by the DNA polymerase Pol*θ* [28]. Finally, evidence suggests that both chromothripsis and replicative errors are not only responsible for highly complex rearrangements involving several chromosomes or a large number of chromosomal segments. Even simpler rearrangements involving a small number of chromosomal segments on a single chromosome could have formed through shattering of a chromosome or replicative errors [21].

To delineate the chromosomes and analyze the plausible underlying mechanisms of formation of multiple CNVs on a single chromosome, we characterized 21 germline complex rearrangements initially detected by CMA. The rearrangements involved only duplications, only deletions or both deletions and duplications. Underlying mechanisms of rearrangement formation were inferred from the BPJ architecture as well as the overall connective picture.

## Results

We investigated the BPs of 21 individuals with clustered germline CNVs using WGS (mate-pair or paired-end sequencing) to elucidate potential underlying mechanisms of rearrangement formation and possibly clinically relevant genomic imbalances or gene disruptions. Cases were included if they harbored two or more CNVs on the same chromosome. The clinical symptoms were variable, including congenital malformations and neurodevelopmental disorders. Phenotypes and CMA results are presented in Table 1.

**Table 1:** Array results and clinical features patients included in the present study.

Case	CMA results ISCN 2016	Pathogenicity	Main reason for CMA referral
**Deletions-only**			
**P2109_190**	arr[GRCh37] 5p15.1(16715952_16736553x1,16758650_16771432x1) NC_000005.9:g.[16715952_16736553del;16736554_ 16758649inv;16758650_16771432del]	VUS	Liver malformation
**P72**	arr[GRCh37] 7q11.22q11.23(70610154_72399292x1,74050199_74834365x1) dn NC_000007.14:g.[70609300_72422999del;72423000_74047984inv;74047986_74049000del]	VUS	Speech delay, Autism
**P2109_302**	arr[GRCh37] 11q14.3(89843044_91294308)x1 mat NC_000011.9:g.[89543002_89640782del;89640783_ 89766001inv;89766002_91339106del]	VUS	Developmental delay, Speech delay, Visual abnormality, Craniosynostosis
**P2109_123**	arr[GRCh37] 17p13.3(2173896_2414920)x1 pat NC_000017.10:g.[2220422_2484969del;2484970_2617882inv;2617882_2649613del]	VUS	Speech delay, ADHD, Autism
**P2109_188**	arr[GRCh37] 21q22.3(43427355_44858483x1,45803409_48095807x1) dn NC_000021.8:g.[43414907_44797114del;44797115_44797221inv; 44797222_45781000del;45781001_45781001inv;45781002_ 48101999del]	Pathogenic	Developmental delay, Speech delay
**P81**	arr[GRCh37] 4q31.3q34.1(155165258_158705411x1,161300937_166372343x1,171349346_174403566x1) dn NC_000004.11:g.[154997276_155050346del;155164913_158707725del;158707726_171342995 inv;161297891_166374443del;171342996_174401004del]	Pathogenic	Developmental delay, Speech delay, Growth retardation
**P2046_133**	arr[GRCh37] 5q31.3q32(144027815_146077337x1,146851376_149511942x1) dn NC_000005.9:g.[389431_146087031delins[154993195_155919592];146087030_146847080inv;146847081_149533960;delins[143779195_144018754inv;141466787_143779194;399867_141466786inv;155929947_157385269];154977468_157385268del]	Likely Pathogenic	Developmental delay, Speech delay
**P00**	arr[GRCh37] 7q11.23q21.11(75063222_77310662x1,77629679_77770664x1,78236090_79911425x1, 82687283_82746799x1)dn NC_000007.14:g.[74942506_77216338delins[77754229_77756619inv;77770732_78236952inv;78265840_82690202inv];77226982_77226980del;77226981_77626463inv;77626464_77626462del;77626463_78265840inv;78265841_82754313del]	Pathogenic	Infantile spasms, Hypotonia
**Duplications-only**			
**P06**	2p25.3(843845_1119040x3, 1611691_1857096x3) mat NC_000002.11:g. [1114148_1114149ins[1610546_1855037;846167 _1114148]] or 2p25.3(843845_1119040x3, 1611691_1857096x3) mat NC_000002.11:g.[1855037_1855038ins[846167 _1114148;1610546_1855037]]	VUS	Developmental delay
**P4855_511**	2p25.3(844930_1112989x3, 1618416_1856851x3) mat NC_000002.11:g.[1114148_1114149ins[1610546_1857566;842609_1114148]] or 2p25.3(844930_1112989x3, 1618416_1856851x3) mat NC_000002.11:g.[1857566_1857567ins[842609_1114148;1610546_1857566]]	VUS	Obesity, Autism, ADHD, Visual abnormality
**P2109_150**	7q31.1(111303881_114362948)x3 mat NC_000007.13:g.[111941768_111941769ins[111963146_114365115;111281787_111941768]] or 7q31.1(111303881_114362948)x3 mat NC_000007.13:g.[114365115_114365116ins[111281787_111941768;111963146_114365115]]	VUS	ADHD, autism
**P2109_151**	arr[GRCh37] 14q32.31(102161711_102573503)x3 mat NC_000014.8:g.[105092354_105092355ins[102138899_102589089inv;104966644_105092354]]	VUS	Psychiatric abnormality
**P74**	16q24.3(88727553_ 89319419x3, 89769750_90022565x3) mat NC_000016.9:g.[90023923_90023924ins[88726889_89324612inv;89772550_90023923]]	Benign	Intellectual Disability, Epilepsy
**P4855_512**	21q22.3(43854701_44578748x3, 44848406_46436410x3) pat NC_000021.8:g.[44845646_44846415_ins43854243_44581164inv;44844321_46454415dup]	VUS	NI
**P5513_206**	14q21.3q31.3(47413346_47731287x3, 49230279_60603652x3,63741627_75994279x3, 86907487_87165260x3) dn NC_000014.8:g.[61179000_61179001ins[47888602_48264000inv;49718081_59901890;87383926_87638698;64300950_76522298inv;59922753_61179000]]	Likely Pathogenic	Heart malformation
**Deletions-and-Duplications**			
**P2109_162**	1q43q44(238817623_244138230x1,245617207_246442209x1,247846701_248592414x3) dn NC_000001.11:g.[238802166_244149898delins[246444835_246492103;247836549_248600189 inv];244149899_246491796inv;245599009_246492102del]	Likely Pathogenic	Microcephaly, Intellectual Disability, Short stature
**P5513_116**	Xp22.33p21.3(285997–26552426)x3,Xq21.1q28(78198636–155559835)x1 dn NC_000023.10:g.[77417096_qterdelins[76868256_77229642inv;pter_26552817inv]]	Likely Pathogenic	NI
**P5371_204**	arr[GRCh37] 13q31.3q34(93528347_110077805x3, 111492168_111972238x1,113582129_114985061x3) dn NC_000013.10:g.[110081347_110102355delins93523111_110075934inv;111492500_111980567del;11358843_115000804dup]	Pathogenic	Developmental delay
**P2109_185**	5p15.33(19,524–2,572,011)x1,5p15.33p14.3(2,556,253–21,131,828)x3,5q35.3(177,638,723–180,712,342)x3 dn NC_000005.9:g.[pter_2559532delins[2587902_7481754inv;177636532_qterinv];7507897_7669625delins7673762_21097826inv]	Pathogenic	Epilepsy
**P2109_176**	2q32.1q36.3(186356601_188906835x1,188926928_225298653x3,225317517_226707110x1) dn NC_000002.11:g.[186345992_186383076delins187132941_186383235; 186383076_186383236inv;186383076_186383235ins[226652944_226738875inv;187132942_187298167];186383236_187298165del;188892330_225311353dup;225311194_226718660del]	Pathogenic	Lung malformation
**P1426_301**	arr[GRCh37] 21q21.1q22.3(16502517_26253075x1,29053919_29464120x1, 33272142_36164839x1, 38469325_38847524x1,27373586_27514060x3,28298721_28571261x3, 31095940_31257111x3, 46317441_46473088x3) dn NC_000021.8:g.[17867977_27624991_delins[29944106_29809107inv;29651577_29785938inv;32467984 _32678337;?;28304789_28316917inv];?_?ins28727001_28879383;30426350_30815784delins30815785_34656669inv;34178763_34656669;34656670_37539019delins[47729066_47896585inv;45504605 _46563358inv;37539020_40225591inv;46546718 _46563358];39830240_40225590del]	Pathogenic	Multiple internal organ malformations, Hypertonia, Visual abnormality (Lindstrand et al., 2010)

mat, maternal; pat, paternal; dn, *de novo*; VUS, variant of uncertain significance; NI, no information

Segregation analysis had been performed in 20 cases and showed that the CNVs were inherited in 8 and *de novo* in 12. Parental DNA samples for further investigation of parental origin were available in seven of the *de novo* cases. It was found that the rearrangement was on the maternal chromosome in four cases and on the paternal chromosome in three cases (S1 Table). We also excluded presence of copy number neutral inversions in the parents. Among the eight inherited cases, the rearrangement segregated from a phenotypically unaffected mother (n = 6) or father (n = 2), indicating that the complex chromosomal rearrangement may be an incidental finding. We detected a complex overall picture with 83 BPs associated with deletions, duplications, inversions and insertions (Table 2; S1 Fig; S2 Table). Resolution was on single nucleotide level in 83 BPJs (75%) (Table 2).

**Table 2:** Characteristics of all breakpoint junctions that were solved on single nucleotide level.

Case	Category	Chromosome	Junction	Side 1	Side 2	Side 1: Repeat	Side 2: Repeat	MH (bp)	Ins (bp)
**P2109_190**	Deletions only	5	1	16715951	16758649	AluSx	MIRb	3	0
			2	16736554	16771433	AluJo	L1P5	0	3
**Patient 72**	Deletions only	7	1	70609299	74047984	LTR26	AluSx	NA	NA
			2	72423000	74049001	L2c	AluSz	NA	NA
**P2109_302**	Deletions only	11	1	89543001	89766001	AT_rich	(TATATG)n	NA	NA
			2	89640783	91339107	SATR1	HAL1	3	0
**P2109_123**	Deletions only	17	1	2220421	2617882	AluSx	AluSx1	32	0
			2	2484970	2649513	AluSq2	AluSq2	NA	NA
**P2109_188**	Deletions only	21	1	43414906	44797221	THE1B	AluSc	0	52
			2	44797115	45781411	AluSc	L1MDa	0	46
			3	45781001	48102000	L1MD2	MLT1I	NA	NA
**Patient 81**	Deletions only	4	1	154997275	155050347	L2b	MER5A1	0	**0**
			2	155164912	171342995	MER81	L1MC2	0	0
			3	158707726	174401005	L2	L1MC4	4	0
			4	161297890	166374444	MSTB	T-rich	NA	NA
**P2046_133**	Deletions only	5	1	389429	154993195	(GGGGA)n	L2a	0	0
			2	399867	155929947	MIR3	AluJr	3	0
			3	141466785	143779195	MER117	(TC)n	0	27
			4	144018754	146087033	L2a	AT_rich	1	0
			5	146847080	155919592	MLT1A0	L1PA7	1	0
			6	149533960	157385269	MIRb	AluJr	0	0
			7	154977468	157385270	MIR	AluJr	0	0
**Patient 00**	Deletions only	7	1	74942505	77756619	(A)n	(TTTA)n	0	3
			2	77216339	79914091	Tigger1	(TG)n	0	0
			3	77226981	77626463	L1MA5	MLT1E1A	1	0
			4	77313213	78267535	Charlie7a	AluJr	1	0
			5	77754229	78236952	(TTTA)n	LTR16E1	NA	NA
			6	77770732	82690202	L2b	MLT1E1	5	0
			7	78265840	82754314	AluY	SVA_B	2	3
**Patient 06**	Duplications only	2	1	846167	1855037	MLT1B	MER31B	NA	NA
			2	1114148	1610546	L1MA7	MLT1K	0	0
**P4855_511**	Duplications only	2	1	842609	1857566	L1MEg	AT_rich	3	0
			2	1114148	1610546	L1MA7	MLT1K	0	0
**P2109_150**	Duplications only	7	1	111281787	114365115	AluSc	L1MA4A	0	NA
			2	111941768	111963146	L2c	L1M4	2	0
**P2109_151**	Duplications only	14	1	102138899	104966644	L1M1	L4	3	0
			2	102589089	105092354	AluSx1	L1MC4a	1	0
**Patient 74**	Duplications only	16	1	88726889	90023923	AluSz6	MLT1K	NA	NA
			2	89324612	89772550	L1M4	MIR	NA	NA
**P4855_512**	Duplications only	21	1	43854243	44846415	MIRb	C-rich	3	0
			2	44581164	44845646	(CA)n	C-rich	2	0
			3	44844321	46454415	AluSc8	(TCCTG)n	2	0
**P5513_206**	Duplications only	14	1	47888602	49718081	AT_rich	L1PA15	0	0
			2	48264000	61179000	L1MEf	AluY	0	0
			3	59901890	87383926	L3	L2	1	0
			4	59922753	64300950	MLT1J	AluSx	0	0
			5	76522298	87638698	Charlie8	AluSc8	2	0
**P2109_162**	Deletions and duplications	1	1	238802165	246444835	L1MD3	L2c	3	0
			2	244149899	246492103	AluJb	L1PA3	2	0
			3	245599008	247836549	L2a	(CATATA)n	5	0
			4	246491796	248600189	AT_rich	AT_rich	2	0
**P5513_116**	Deletions and duplications	X	1	26552817	76868256	L2c	L1MB4	2	0
			2	77229642	77417095	L1M5	L1PBa1	NA	NA
**P5371_204**	Deletions and duplications	13	1	93523111	110102355	MIRb	(TA)n	0	8
			2	110075934	110081348	L3	L3	2	0
			3	111492499	111980568	L1M4	LTR38B	2	0
			4	113588473	115000804	MER5A	L1MC4a	2	0
**P2109_185**	Deletions and duplications	5	1	2559532	2587902	(T)n	MLT1E1A	0	17
			2	7481754	177636532	L1MA3	MIRb	1	0
			3	7507896	21097826	MIR	LTR67B	0	1
			4	7669627	7673762	MER112	MER20	0	12
**P2109_176**	Deletions and duplications	2	1	186345992	187132941	L2	L1PA7	NA	NA
			2	186383076	226738875	L1PA8	L1PA2	5	0
			3	186383301	187298167	L1P3b	HERVL18-int	0	42
			4	186383200	188892000	L1PA8	L1PB1	NA	NA
			5	186383235	187133023	L1P3b	L1PA7	NA	NA
			6	187132942	226652944	L1PA7	AluJr	4	52
			7	188892330	225311353	L1PB1	L1MEg	3	1
			8	225311193	226718661	L1MEg	L1PA2	4	0
**P1426_301**	Deletions and duplications	21	1	17867977	29944106	AT_rich	(TTATA)n	0	2
			2	27624991	28304789	L2c	AluSg	1	0
			3	29651577	32467984	MIR	L1PA15	0	8
			4	29785938	29809107	AluY	(TTTA)n	0	23
			5	30426349	34185841	AluY	AT_rich	0	14
			6	30815785	34656669	L1PA2	AluSq	2	0
			7	34178503	47896585	LTR88a	AluSz	NA	NA
			8	37539020	46546718	MER1B	MIR3	2	0
			9	39830239	45423086	AluSg	AT_rich	2	0
			10	40225591	46563358	MIRb	L2a	3	0
			11	45504605	47729066	MER21B	AluSx	NA	NA
			12	28879383	NA	L1MA8	NA	NA	NA
			13	28316917	NA	L2a	NA	NA	NA
			14	32678337	NA	L1MC4	NA	NA	NA

Details of microhomology and inserted sequences are provided in S2 Table. MH, microhomology; Ins, insertion; NA, not applicable

In ten cases, two distinct patterns DEL-INV-DEL (n = 4) and DUP-DIP-DUP (n = 6) were observed (DEL, deletion; INV, inversion; DUP, duplication; DIP, diploid). In four of these (P2109_302, P2109_123, P2109_150, P2109_151), the initial CMA suggested a single deletion or duplication and the nature of the rearrangement was resolved with WGS (Table 3). The remaining 11 cases showed unique patterns (Table 3).

**Table 3:** Copy number status and fragment orientation as revealed by chromosomal microarray (CMA) and whole genome sequencing (WGS) of the complex rearrangements.

Case	CMA results	WGS results
**Deletions-only**		
**P2109_190**	DEL-DIP-DEL	DEL-INV-DEL
**P72**	DEL-DIP-DEL	DEL-INV-DEL
**P2109_302**	DEL	DEL-INV-DEL
**P2109_123**	DEL	DEL-INV-DEL
**P2109_188**	DEL-DIP-DEL	DEL-INV-DEL-INV-DEL
**P81**	DEL-DIP-DEL-DIP-DEL	DEL-N-DEL-INV-DEL-INV-DEL
**P2046_133**	DEL-DIP–DEL	DEL-INV-INV-INV-DEL-INV-DEL-N-DEL-N-DEL-N
**P00**	DEL-DIP-DEL-DIP-DEL-DIP-DEL	DEL-INV-DEL-INV-DEL-INV-DEL-INV-DEL-INV-DEL-N-DEL
**Duplications-only**		
**P06**	DUP-DIP-DUP	DUP-N-DUP
**P4855_511**	DUP-DIP-DUP	DUP-N-DUP
**P2109_150**	DUP	DUP-N-DUP
**P2109_151**	DUP	DUPinv-N-DUP
**P74**	DUP-DIP-DUP	DUP-N-DUP
**P4855_512**	DUP-DIP-DUP	DUPinv-N-DUP
**P5513_206**	DUP-DIP-DUP-DIP-DUP-DIP-DUP	DUPinv-N-DUP-N-DUP-N-DUPinv-N-DUP
**Deletions-and-duplications**		
**P2109_162**	DEL-DIP-DEL-DIP-DUP	DEL-INV-DEL-N-DEL-N-DUP
**P5513_116**	DUP-DIP-DEL	DUP-N-DUP-N-DEL
**P5371_204**	DUP-DIP-DEL-DIP-DUP	DUP-N-DEL-N-DEL-N-DUP
**P2109_185**	DEL-DUP-DEL-DUP-DIP-DUP	DEL-N-DUP-DEL-N-DUP-N-DUP
**P2109_176**	DEL-DUP-DEL	DEL-INV-DEL-N-DEL-DUP-DEL-DUP
**P1426_301**	DEL-DIP-DUP-DIP-DUP-DIP-DEL-DIP-DUP-DIP-DEL-DIP-DEL-DUP	DEL-N-DUP-N-DUP-N-DUP-N-DUP-N-DEL-INV-DUP-INV-DUP-N-DEL-N-DEL-N-DUP-N-DUP-N-DUP

N, normal; DIP, diploid; DUP, duplication; DEL, deletion; DUPinv, inverted duplication; INV, inversion; CMA, chromosome microarray, WGS, whole genome sequencing

### Classification of complex clustered CNVs

**Fig 1:**
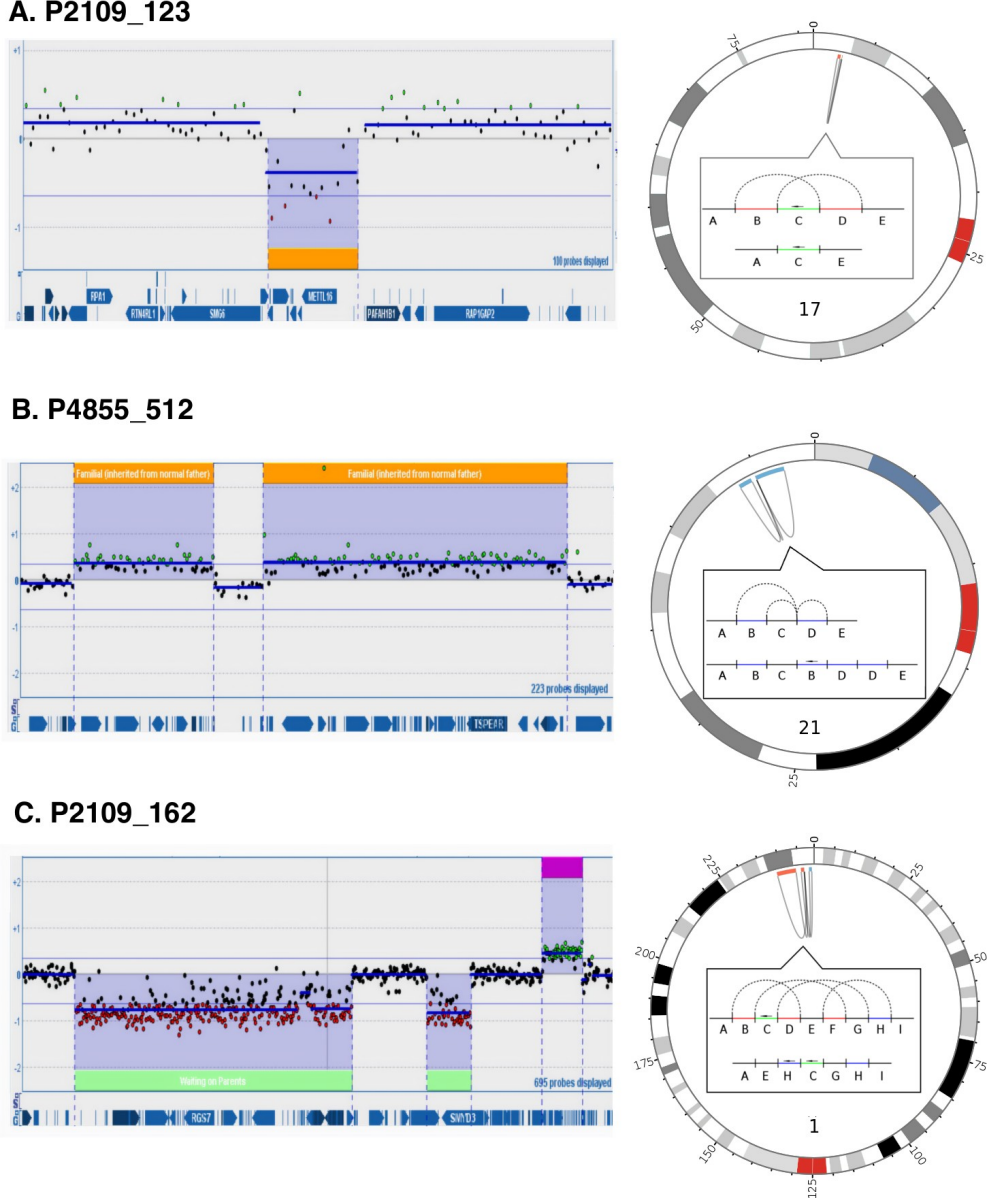
Schematic illustrations of WGS results from three cases representing the three complex CNVs categories: (1) deletions only, (2) duplications only, and (3) deletions and duplications. (A) Case P2109_123 with DEL-INV-DEL, (B) Case P4855_512 with DUP-N-DUP, and (C) Case P2109_162 with a complex rearrangement consisting of inversions, deletions and duplications (DEL-INV-DEL-N-DEL-N-DUP). For case P2109_123 the array-CGH analysis only identified a single deletion and the complex rearrangement was only seen by the WGS analysis. For all the array-CGH results are visualized as a plot seen on the left. The individual dots represent specific oligonucleotide probes and are indicated as black (normal copy number), green (copy number gain), and red (copy number loss) compared to a reference sample. Genes are shown as blue arrows below. On right side the WGS result is shown, illustrated as a Circos plots and within the Circos plots as linear plot with copy number status indicated as black (normal copy number), blue (copy number gain), or red (copy number loss) and inverted segments marked with an arrow. Linked reads showing connections between chromosomal BPs are illustrated as dashed lines.

Based on the CNV type, all rearrangements were classified into deletions-only group (n = 8), duplications-only group (n = 7) and deletions-and-duplications group (n = 6) (S1 Fig). Examples from each group are presented in Fig 1. The average number of BPJs per case was 4 (range = 2–14). The rearrangements in the duplications-only group contained the fewest BPJs per case (average = 3, range = 2–5) and consisted mostly of DUP-DIP-DUP rearrangements (Table 1). The rearrangements in the deletions-only group contained slightly more junctions (average = 4, range = 2–7). The rearrangements belonging to the deletions-and-duplications group showed the highest degree of complexity with more BPJs per case (average = 6, range = 2–14).

### Clustered CNVs show additional complexities at nucleotide-level resolution

**Fig 2:**
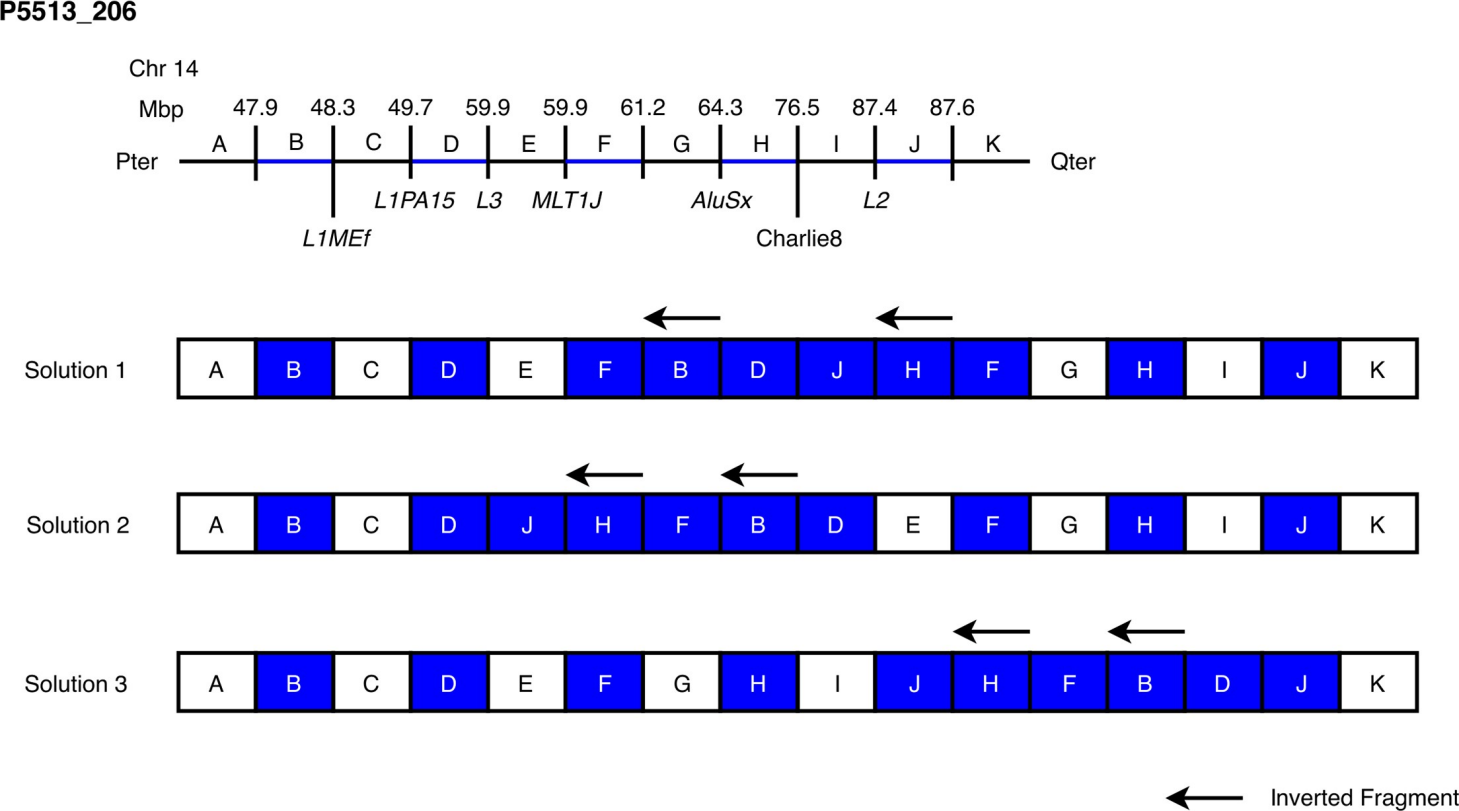
Three different plausible end products in a complex case involving five duplications. In case P5513_206, five duplications were shown to not be tandem, but inserted in a seemingly random but clustered manner. The exact location of each duplicate could not be determined using WGS only, but three plausible outcomes are shown. Here we show a schematic drawing of the 11 chromosomal segments involved on human chromosome 14q labelled A-K. In the linear representation the copy number status is indicated as black (normal) or blue (duplicated). Each BP is shown as a short vertical black line. Above the line the genomic coordinates of identified BPs is indicated and if repeat elements are disrupted by a BP they are shown below the line. In the three solutions the regions are shown as boxes and copy number status is indicated as white (normal) and blue (duplicated).

**Fig 3:**
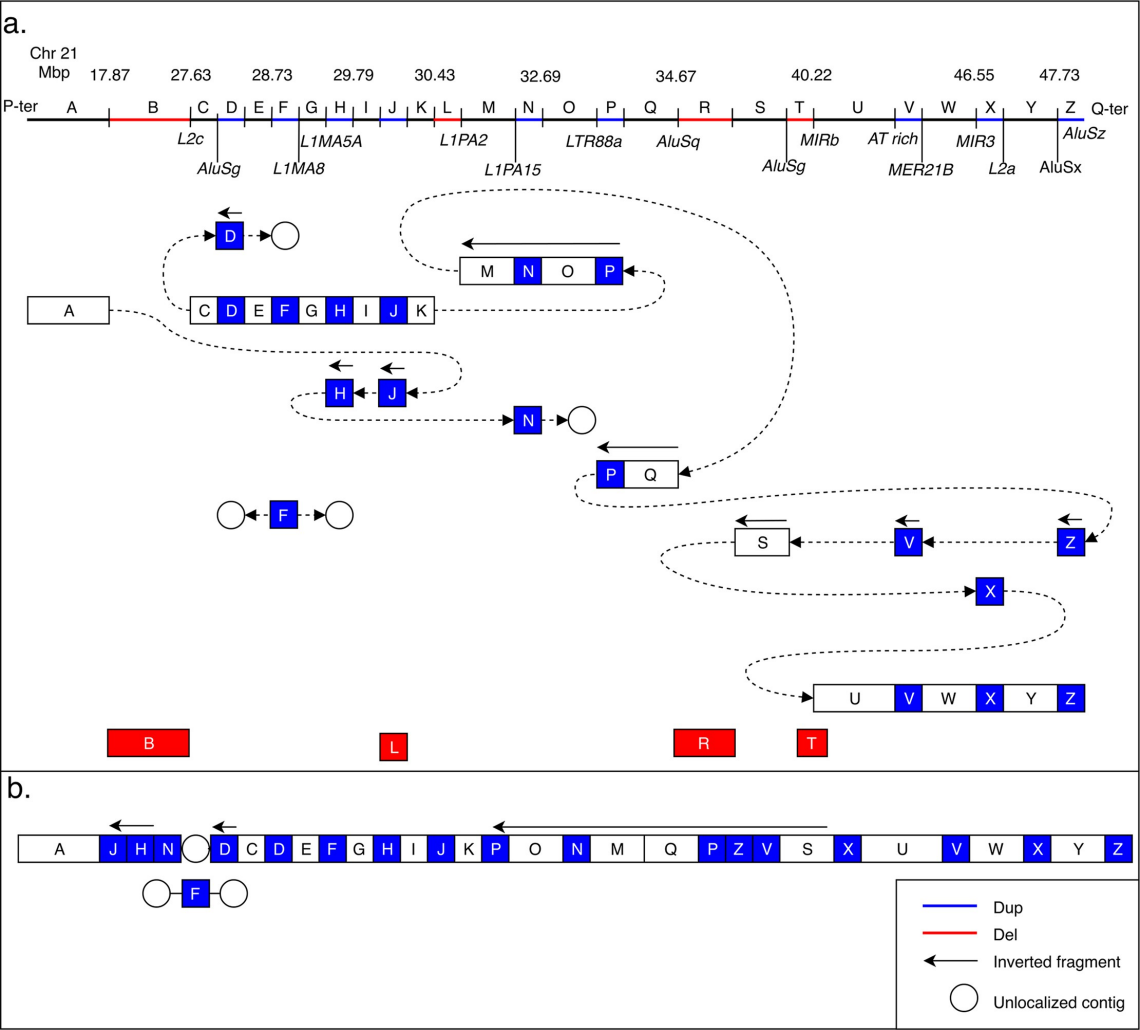
A schematic picture of the complex rearrangement of chromosome 21 involving deletions, duplications, and inversions in case P1426_301. On top is a connectivity diagram (A). The upper bar indicates the position and copy number of the fragment (blue for duplication, and red for deletion) as well as repeats elements found at the BPs. Below, each box illustrates a fragment involved in the rearrangement (A-Z). The circles represent contigs that are not positioned within GRCh37/hg19, as well as poorly defined centromeric regions. The lines connecting the boxes and circles illustrate the fusion of the various fragments. At the bottom (B) is a diagram of the final derivative chromosome. It is not certain where the duplicate of fragment F is inserted.

In total, WGS revealed additional duplicated or deleted fragments not detected by CMA in 16 out of 21 cases (76%) (Table 3). In most of the cases, the obtained BPJs allowed us to resolve the exact nature of rearranged chromosomes. For one case (P5513_206) from the duplications-only group, there was no conclusive order for the duplicated fragments, hence three possibilities are shown in Fig 2. In one highly complex case (P1426_301) the full connective picture of rearranged chromosomes could not be established (Fig 3).

In four cases where CMA suggested two clustered duplications separated by a diploid fragment (P4855_511, P2109_150, P06 and P74), WGS revealed a nested deletion within the duplicated segment (S2 Fig). Notably, all these four rearrangements were maternally inherited indicating that the duplication and the deletion are located in *cis*. In addition, WGS allowed detection of copy-neutral segments (inversions and insertions); and in total, 37 inversions were detected within the clustered CNVs (Table 3). The deletions-only group contains a large number of inverted fragments similar to the deletions-and-duplications group, while the duplications-only group contains only four duplicated fragments with inverted orientation in three cases (P209_151, P4855_512 and P5513_206) (Table 3).

### Additional disease causing genes were revealed by WGS

Several OMIM morbid genes were identified in clustered CNVs detected by CMA (S3 Table). A CNV was assessed as pathogenic or likely pathogenic in 11 cases, as benign in one case, and in the remaining cases as variants of unknown significance (Table 1). The pathogenicity classification was based on the American College of Medical Genetics and Genomics (ACMG) guidelines [29] and included the segregation analysis, amount of OMIM morbid genes or specific disease-related genes, size of the CNVs and/or if the CNVs had been reported previously in patients with similar phenotype. None of the CNVs disrupted an OMIM morbid gene but all CNVs that were classified as likely pathogenic or pathogenic was based on gene dosage sensitivity mechanisms. In four cases (P2046_133, P5513_206, P5513_116 and P1426_301) WGS enabled detection of further OMIM morbid genes, which could not be revealed by CMA (S3 Table).

### Duplications are mostly interspersed and not tandem

Thirteen of the 21 rearrangements consisted of 36 duplicated fragments (Table 1): 17 of these fragments belong to the duplications-only group (7 individuals) and 19 fragments belong to the deletions-and-duplications group (6 individuals). In all cases, the WGS data analysis could detect whether the duplications were tandem (3 fragments) or interspersed (33 fragments).

Notably, the majority of the duplications were interspersed (92%). There was a single tandem duplication in the duplications-only group (P4855_512) and two tandem duplications in the deletions-and-duplications group (P5371_204 and P2109_176) (Fig 1B). All interspersed duplications were intrachromosomal and 46% of the duplicated fragments were inverted, indicating random orientation of the duplicates. The duplicates of the interspersed duplications clustered tightly: 79% of the duplicates were inserted next to another duplicate. P5513_206 represents such a rearrangement that consists of five interspersed duplications, all inserted in a clustered but seemingly random manner in the same region (Fig 2).

### Breakpoint junction characteristics

**Fig 4:**
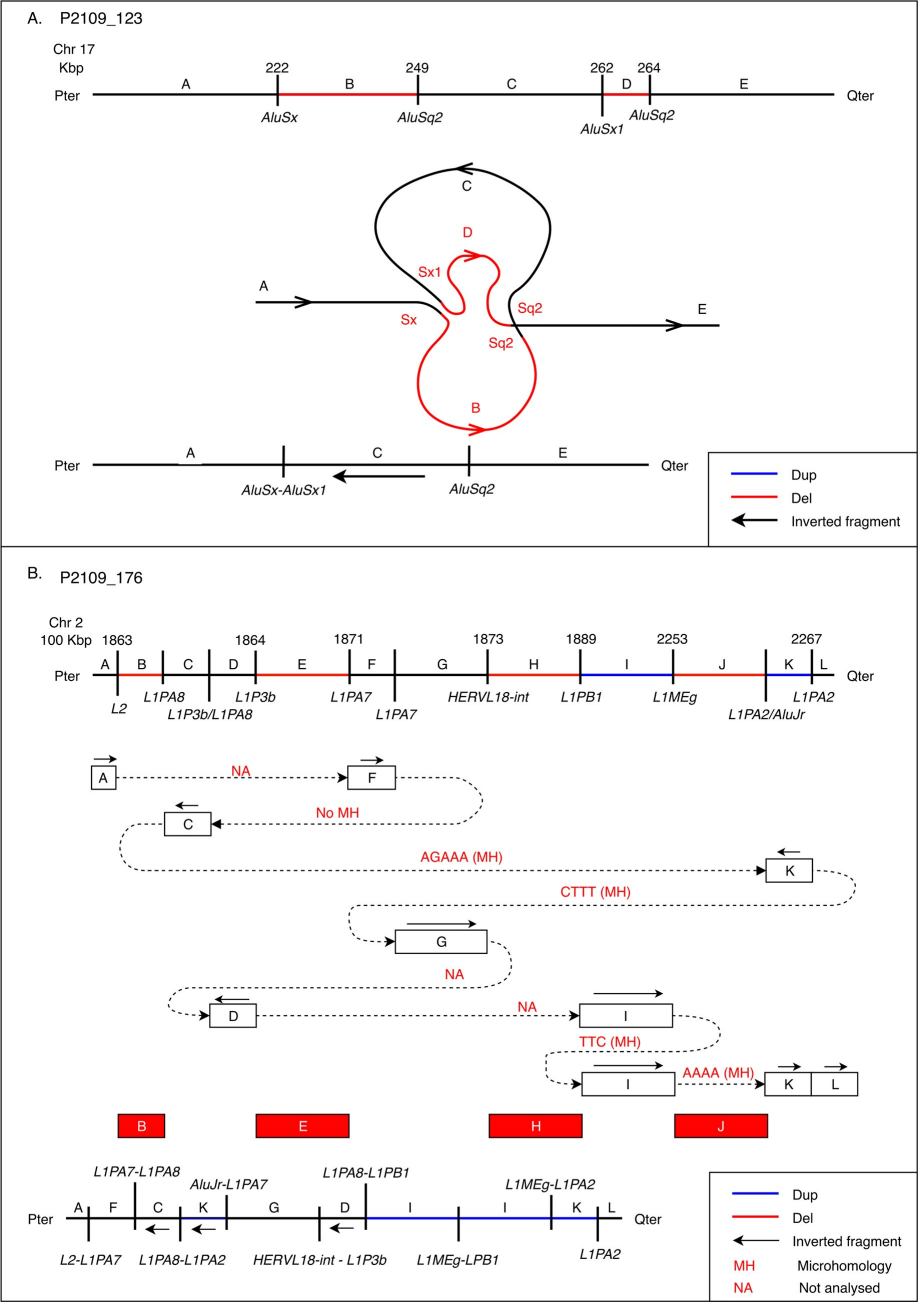
A schematic picture of *Alu*-*Alu* and LINE-mediated rearrangements. **(**A) Case P2109_123 states as an example of an *Alu*-mediated DEL-INV-DEL rearrangement. Copy number status is indicated as black (normal copy number) or red (copy number loss), and inverted segments marked with an arrow. Repeat elements located at the BPs junctions are indicated. In BPJ A-C, an *Alu* fusion seem to have formed. B) Case P2109_176 represents LINE-mediated rearrangements. On top is a connectivity diagram. The upper bar indicates the position and copy number of the fragment (blue for duplication, and red for deletion) as well as LINE elements found at all the BPs. Below, each box illustrates a fragment involved in the rearrangement (A-L). The lines connecting the boxes illustrate the fusion of the various fragments, and microhomology is shown on top of connections whenever it was detected (NA: not analysed). At the bottom is a diagram of the final derivative chromosome.

Of the 83 total BPJs, 63 (19 cases) were resolved to single nucleotide resolution (Table 2). SplitVision analyses suggested the following features for the BPJs: novel single nucleotide variants (SNVs) within 1 kb of the BPJ (absent in gnomAD and SweFreq), microhomology, short insertions and repeat elements. Most of the rearrangements contained at least one of these features (S2 Table, Table 2). In total, 30 BPJs (48%) contained microhomology stretches ranging from 2 to 32 nucleotides (median = 2) (S2 Table, S5 Fig, S6 Fig). Even though repeat elements were enriched in BPJs, fusions of similar repeats were only observed in 11 BPJs (13%). The longest stretch of microhomology was 32 nucleotides (P2109_123) and involved homologous *Alu* associated BPs (Fig 4A). Similarly, all the 11 BPs in P2109_176 contained LINE elements resulting in fusion LINEs at the BPJs (Fig 4B). The most complex case, P1426_301, contained deletions, duplications, and inversions and harbored 25 BPs (14 BPJs) where 16 (64%) were located within repeat regions (Fig 3, S6 Fig). In two cases (P4855_512 and P5371_204), two BPJs harbored novel SNVs within 1 kb of BPJs localized to non-coding regions. Lastly, 10 blunt BPJs were identified in 5 cases (P2046_133, P81, P00, P4855_511, P06) (Table 2, S2 Table, S6 Fig). P2046_133, P81 and P00 belong to the deletions-only group, and P4855_511 and P06 belong to the duplications-only group. No blunt BPJs were found in the deletions-and-duplications group (Table 2). Comprehensive analysis of the BPJ characteristics surrounding the BPJs in all cases and comparisons between the groups are presented in S5 Fig and S6 Fig.

### Mutational signatures indicating underlying mechanisms of rearrangement formation

Molecular signatures at the BPJs further enabled the reconstruction of underlying mutational mechanisms. For example, blunt joints, absent or short microhomology (1–4 bp) and small insertions or deletions at the BPJs are characteristic of DNA DSB repair through direct ligation by NHEJ. In the clustered CNVs studied here, we observed that most of the BPJs involved in the deletions-only group showed such signatures (Table 2, S2 Table) pinpointing involvement of NHEJ. Alternatively, DNA DSBs can also be repaired by alternative NHEJ (alt-NHEJ) mechanisms, such as MMEJ which is a more error prone repair pathway highly dependent on microhomology [17]. MMEJ may result in deletions of the DNA regions flanking the original BP, and longer stretches of both templated (sequences found within 100 nucleotides upstream or downstream of the junction) and non-templated (seemingly random nucleotides) insertions at the BPJs. One of the characterized BPJs in P2109_188 has very typical signatures of MMEJ: a 14bp non-templated insertion followed by a 26 bp templated insertion (chr21:45466217–45466242, (-) strand), followed by another 12 bp non-templated insertion, plus 3 bp and 4bp microhomologies at the 5’- and the 3’-sides of the BPJ (S3 Fig). Short stretches of microhomologies (2–3 bp) were also found at other BPJs in the deletions-only group (i.e. P00, P2046_133, P2109_190, P2109_302). It is important to note that these features are also overlapping with features consistent with alt-NHEJ mediated by PARP1, CTIP, MRE11, DNA ligase I/III and polymerase θ (Polθ) [28,30,31], which is associated with short single-strand overhangs after a DSB. This typically leads to inserts of 5–25 bp before ligation and hence leads to short stretches of microhomology seen in the BPJ [31], similar to what is seen in MMEJ. In addition, canonical NHEJ and alt-NHEJ can operate simultaneously in the same cell [32], and this possibility needs to be taken into consideration as well.

Overall, microhomologies were mostly prevalent at the BPJs of the complex rearrangements containing duplications (54% and 59% for duplications-only group and deletions-and-duplications group, respectively) (Table 2, S5 Fig). A model of replication-based mechanisms, for example multiple template switching, could better explain the formation of these complex rearrangements (Fig 3B, Fig 4). Such mechanisms are commonly associated with similar features as MMEJ, as well as *de novo* single nucleotide variants around the BPJs [33].

### Identical rearrangements on 2p53.3 in two unrelated individuals

Seemingly identical rearrangements on 2p25.3 were identified in individuals P4855_511 (from Sweden) and P06 (from Denmark), belonging to the duplications-only group based on CMA results. However, these two cases were later redefined as having duplication with a “nested” deletion inside the duplicated fragment. An identical blunt BPJ without microhomology (the BPJ of the nested deletion) was detected in both P4855_511 and P06. The duplication junction was resolved at nucleotide level only in P4855_511 and a 3bp microhomology (TGC) was detected at the BPJ through split reads in the deep paired-end data. However, for case P06 no split-read was present for the BPJ showing the duplication in the shallow mate-pair WGS data. Several attempts were made to amplify the BPJ using breakpoint PCR and Sanger sequencing without success due to GC-rich sequences in the area. Hence, we could only compare the junction sequences of one junction, which were identical, including a SNV (rs4971462) in *cis* upstream of the junction (S4 Fig). This may suggest that the 2p25.3 could be a rare founder variant in Europe. However, using the WGS data from P4855_511 and the Affymetrix Cytoscan HD SNP array data from P06, we analyzed 100 common SNVs surrounding the rearrangement and found that the haplotypes for these variants varied in a way that would be expected for two unrelated individuals. Hence, it was not possible to assess whether the rearrangement in these two individuals have occurred through separate events or in a common ancestor. No evidence suggest that the region is a hotspot for CNV formation, no common repeat structure was present in the BPJs and we also assessed the junction sequence from the common BPJ (S4 Fig) in the Predict a Secondary Structure Web Server (https://rna.urmc.rochester.edu/RNAstructureWeb/Servers/Predict1/Predict1.html) and no significant structure was seen. Remaining rearrangements were all unique.

Finally, the junction architecture may indicate that the nested deletion occurred via non-replicative mechanisms (e.g. NHEJ), which require no microhomology. Although the tandem duplication might occur during replication process, we hypothesize that they occurred within a single cell cycle, as the duplication is co-segregated with deletion in both families.

### *Alu*-*Alu* and LINE mediated rearrangements

We and others have previously shown that the sequence homology between *Alu* elements (average 71%) may facilitate unequal crossover between genomic segments and generate *Alu*-*Alu* mediated CNVs, inversions, translocations and chromothripsis [15,34,35]. In the current cohort, DEL-INV-DEL rearrangements on 17p13.3 are associated with fusion *Alu–Alu* elements at both junctions (P2109_123), suggesting an *Alu-Alu* mediated mechanism in this complex rearrangement. Sequence identity between the *AluSx*_*AluSx1* and *AluSq2_AluSq2* pairs are 73.3% and 78.6%, respectively. Notably, both *AluSx*_*AluSx1* and *AluSq2_AluSq2* pairs are in opposite orientation on the reference genome, which resulted in inversion of the fragment C (Fig 4A). As the sequence identity of involved *Alu* pairs is < 90%, it might not be sufficient for homologous recombination, while MMEJ or FoSTeS/MMBIR could potentially generate *Alu-Alu* mediated rearrangements here as previously suggested by other studies [34–36]. Indeed, 17p13.3 region is known to be *Alu* rich and consequently many *Alu-Alu* mediated CNVs and complex genomic rearrangements associated with multiple disorders have been reported [35]. Similarly, in P2109_176 involving a combination of deletions, duplications and other copy-neutral rearrangements on chromosome 2, we observed LINE elements at all 11 BPs, indicating underlying LINE-mediated mechanisms (Fig 4B). Here, we found 3–5 bp microhomologies at most of the BPJs, indicating replication based FoSTeS/MMBIR mechanisms likely being involved in this case.

Finally, 14 out of 25 BPs in the most complex case (P1426_301) containing deletions, duplications, and inversions are located within repeat regions of different classes likely providing microhomology for multiple template switching (Fig 3).

## Discussion

In the current study we present 21 individuals with two or more clustered non-recurrent CNVs confined to a single chromosome including both chromosomal arms (two cases) or to a single chromosomal arm (19 cases). WGS enabled us to decipher the true nature of the rearrangements including detection of copy neutral variants within or flanking the rearrangements. The individuals had a wide range of clinical symptoms, including congenital malformations and neurodevelopmental disorders. Dosage of the genes located within the deleted and/or duplicated fragments and/or the disruption of genes located in the BPJs could be responsible for the clinical manifestations. In the current cohort, the more exact resolution of WGS as compared to CMA resulted in a reduction of the number of morbid OMIM genes affected in three cases (14%) and in an increase in one individual (5%). However, this information did not influence the overall assessment of the clinical relevance.

WGS analysis revealed additional complexities such as inversions and interspersed duplicates in most cases, findings that are in line with previous findings in a cohort of autism spectrum disorder where 84.4% of large complex SVs involved inversions [3]. In addition, we detected that most of the interspersed duplications were inserted next to another in a seemingly random manner, similar to the few cases reported before [28].

For ultra-complex chromosomal rearrangements such as the ones seen in P1426_301 and P00, the large number of genomic pieces with breakpoints often located in repetitive regions complicates the mapping of the final structure of the derivative chromosome(s). Third-generation sequencing including Pacific Biosciences SMRT long-read sequencing platform or Nanopore MinION sequencing has showed promising results [37,38] for bridging repetitive sequences and hence overcoming one of the largest limitations with short-read sequencing. The current study is limited by the fact that we did not try any of these technologies, which would be the next step needed to completely solve the structure of the derivative chromosomes in this case (P1426_301). Long-read sequencing might also add information in case P5513_206 that is presented here with three possible rearrangements of the duplicated fragments.

By mapping all the BPs and resolving the links between the generated fragments, we observed several hallmarks of germline chromothripsis and chromoanasynthesis [4,25,39]. First, all the BPs associated with the complex rearrangements were clustered and confined to a single chromosome. Second, the rearranged fragments within the derivative chromosomes had random order and orientation. Third, the copy-number states detected in deletions-only group oscillated between one and two, typical to chromothripsis, while the rearrangements including duplications were mostly resembling chromoanasynthesis. Fourth, signatures of NHEJ and MMEJ pathways were mostly detected at the BPJs of the complex rearrangements included in the deletions-only group, which is compatible with the previous reports describing BPJs associated with chromothripsis [9,18,19,32]. Even though both chromothripsis and chromoanasynthesis are generally of paternal origin [6,40], the current *de novo* chromosomal rearrangements occurred on the maternal and paternal chromosomes to the same extent. Of the seven *de novo* cases where we had parental samples, three had characteristics of chromoanasynthesis and replicative errors and two of those arose on the maternal chromosome. This is in contrast to the expectation that replicative error-mediated chromosomal aberrations would be biased towards spermatogenic origin. In addition, among the four cases with characteristics of chromothripsis, two were of paternal origin and two of maternal origin. Finally, we confirmed that *Alu-* or LINE- mediated mechanisms may also underlie chromothripsis formation.

Most of the reported germline chromothripsis cases are nearly dosage-neutral, possibly due to embryonic selection against loss of dosage-sensitive genes. However, there are few reports of heavy imbalances detected by CMA, suggesting chromothripsis event [41–45]. Such cases need further investigations by paired-end or mate-pair sequencing in order to decipher the balanced rearrangements involved as well as to understand the underlying mechanisms. Our approach of applying high-resolution sequencing in such cases with clustered deletions, confirmed that additional copy-neutral SVs may coexist. Combined picture of such complex rearrangements resembled catastrophic phenomenon of chromosome “shattering”, where some of the fragments may be lost (deleted), while retained fragments would be resembled by repair machinery with random order and orientation. The fact that clustered duplications and combinations of deletions and duplications typical to chromoanasynthesis revealed both non-tandem and inverted nature of most duplicates, enriched with microhomologies at the BPJs, further supports the notion that replication based mechanisms, may explain the complex nature of these derivative chromosomes. In summary, we suggest that seven cases in the current study (P2109_190, P72, P2109_302, P2109_123, P2109_188, P81 and P00) represents chromothripsis, ten cases (P06, P4855_511, P2109_150, P2109_151, P74, P4855_512, P5513_206, P2109_162, P5513_116, P5371_204) are chromoanasynthesis events and four cases (P2109_185, P2109_176, P2046_133 and P1426_301) have ambiguous mutational signatures. All four ambiguous cases showed large non-templated insertions in the BPJ (typical to Polθ-driven atypical chromoanagenesis or retrotransposition-mediated chromothripsis), but three cases harbored both duplications and deletions (typical to chromoanasynthesis) and one case contained only deletions (typical to chromothripsis). Of the seven chromothripsis cases, one case was *Alu-Alu* mediated (P2109_123) and one was likely mediated by replicative errors and the DSBs were joined through alt-NHEJ (P2109_188), while remaining cases showed more consistent signatures of canonical NHEJ or MMBIR. Among the cases involving duplications or both duplications and deletions, most BPJs showed signatures of replicative errors with microhomology in the breakpoints, some possibly caused by repeat elements, except in three cases from the deletions and duplications-group (P2109_185, P2109_176, P1426_301) with non-templated insertions ranging in 8–52 bp in size and short microhomology (2–6 nt) in the BPJs. These features are not fully consistent with replicative joining mechanisms such as FoSTeS/MMBIR, but it is possible that these cases are mediated by replicative errors, and that Polθ is involved in the stitching of the chromosomes, hence two operating repair machineries in the same cell.

In two of the cases in our cohort (P5513_116 and P2109_185) the clustered CNVs were detected on both arms of the chromosomes involved (chromosome X and 5, respectively). Notably, these two cases show similar patterns, where a terminal duplication of one chromosomal arm is inserted in the place of terminal deletion of the other chromosomal arm with an inverted orientation. A breakage-fusion-bridge cycle process could explain parts of this kind of rearrangement. Briefly, the process starts when a chromosome loses its telomere and after replication the two sister chromatids will fuse into a dicentric chromosome [46]. Then, during anaphase the two centromeres will be pulled towards opposite nuclei, resulting in the breakage of the dicentric chromosome. Random breakage may cause large inverted duplications. After the breakage there will be new chromosome ends lacking telomeres resulting in a new cycle of breakage-fusion-bridge, the cycles will stop once the chromosome end acquires a telomere. This mechanism has previously been suggested to explain some cases of chromothripsis formation [9,13,47]. Here, with telomeric regions of both chromosome arms being involved, it is likely that the breakage-fusion-bridge cycle has been accompanied by a formation-attempt of a ring chromosome. However, chromosome analysis and FISH had previously shown that no ring chromosome was formed in either of these cases. In addition, as mentioned previously, case P2109_185 showed characteristics of Polθ involvement in the stitching with large non-templated insertions in the BPJs.

In conclusion, the BP characterization of the derivative chromosomes showed that multiple mechanisms are likely involved in the formation of clustered CNVs, including replication independent canonical NHEJ and alt-NHEJ, replication-dependent MMBIR/FoSTeS and breakage-fusion-bridge cycle, as well as *Alu*- and LINE-mediated pathways. WGS characterization adds positional information important for a correct interpretation of complex CNVs and for determining their clinical significance; and deciphers the mechanisms involved in formation of these rearrangements.

## Methods

### Ethics statement

The local ethical board in Stockholm, Sweden approved the study (approval number KS 2012/222-31/3). This ethics permit allows us to use clinical samples for analysis of scientific importance as part of clinical development. Included subjects were part of clinical cohorts investigated at the respective centers and the current study reports de-identified results that cannot be traced to a specific individual. All subjects have given oral consent to be part of these clinical investigations.

### Study cohort

The subjects included in this study (n = 21) were initially referred to the Department of Clinical Genetics at the Karolinska University Hospital (n = 13), Kennedy Center (n = 5), Sahlgrenska University Hospital (n = 2) or Linköping University Hospital (n = 1). All subjects were part of clinical cohorts investigated at respective centers with CMA due to congenital developmental disorders, intellectual disability or autism. Karyotypes and phenotypes are provided in Table 1.

### Chromosome microarray analysis

Genomic DNA was prepared from whole blood using standard procedures. CMA was carried out using either SNP (single nucleotide polymorphism) or oligonucleotide microarrays. Fluorescent *in situ* hybridization (FISH) analysis or quantitative PCR (qPCR) with Power SYBR Green reagents (Applied Biosystems, Carlsbad, CA, USA) was employed to verify the structural variants. FISH-, qPCR-, or array comparative genomic hybridization (aCGH) analysis was used to investigate parental inheritance when possible.

In 13 cases (P2046_133, P2109_123, P2109_150, P2109_151, P2109_162, P2109_188, P2109_190, P2109_302, P4855_511, P4855_512, P2109_176, P1426_301, P2109_185), the CMA was performed with an 180K custom oligonucleotide microarray with whole genome coverage and a median resolution of approximately 18 kb (Oxford Gene Technology (OGT), Oxfordshire, UK). Experiments were performed at the Department of Clinical Genetics at Karolinska University Hospital, Stockholm, Sweden, according to the manufacturer’s protocol. Slides were scanned using an Agilent Microarray Scanner (Agilent Technologies, Santa Clara, CA, USA). Raw data were normalized using Feature Extraction Software (Agilent Technologies, Santa Clara, CA, USA), and log2 ratios were calculated by dividing the normalized intensity in the sample by the mean intensity across the reference sample. The log2 ratios were plotted and segmented by circular binary segmentation in the CytoSure Interpret software (OGT, Oxfordshire, UK). Oligonucleotide probe positions were annotated to the human genome assembly GRCh37 (Hg19). Aberrations were called using a cut-off of three probes and a log2 ratio of 0.65 and 0.35 for deletions and duplications, respectively.

For eight cases (P72, P81, P06, P74, P5513_206, P5513_116, P5371_204, P00) the CMA was performed using an Affymetrix CytoScan HD array and data were analyzed with ChAS software (Affymetrix, Santa Clara, CA, USA) using the following filtering criteria: deletions > 5 kb (a minimum of 5 markers) and duplications >10 kb (a minimum of 10 markers). Patients’ CNV data were reported to ClinVar (P2046_133, P2109_123, P2109_150, P2109_151, P2109_162, P2109_188, P2109_190, P2109_302, P4855_511, P4855_512, P2109_176, P1426_301, P2109_185, P5513_206, P5513_116, P5371_204) or to DECIPHER (P72, P81, P06, P74, P00).

### Mate-pair WGS

Mate-pair libraries were prepared using Nextera mate-pair kit following the manufacturers’ instructions (Illumina, San Diego, CA, USA). The subjects were investigated with the gel-free protocol where 1 μg of genomic DNA was fragmented using an enzymatic method generating fragments in the range of 2–15 kb. The final library was subjected to 2x100 bases paired-end sequencing on an Illumina HiSeq2500 sequencing platform.

### Paired-end WGS

The PCR-free paired-end Illumina WGS data was produced at the National Genomics Infrastructure (NGI), Stockholm, Sweden. The WGS data was generated using the Illumina Hiseq Xten platform, which produced an average coverage of 30X per sample. The average insert size of the WGS libraries was 350 bp, and each read length was 2x150 bp.

### WGS analysis

The WGS data was aligned to GRCh37 (Hg19) using BWA-mem (version 0.7.15-r1140) [48], and duplicates were marked using Picard tools (http://broadinstitute.github.io/picard/). Structural variant calling was performed using FindSV (https://github.com/J35P312/FindSV), which combines CNVnator [49] and TIDDIT [50]. The variant call format (vcf) files of these two callers were merged and annotated using VEP [51] and filtered against an internal frequency database consisting of 350 individuals. The exact position of the BPs was pinpointed using split reads (S2 Table; cases P2046_133, P2109_123, P2109_150, P2109_151, P2109_162, P2109_188, P2109_190, P2109_302, P4855_511, P4855_512, P2109_176, P5513_116, P5371_204, P1426_301, P2109_185) or Sanger sequencing (cases P00, P06 and P81; Primers and PCR conditions will be provided upon request).

The WGS data and Sanger reads were analyzed for junction features such as microhomology, insertions, single nucleotide variants (SNVs), and repeat elements using blat (https://genome.ucsc.edu/cgi-bin/hgBlat?command=start) and an in-house developed analysis tool dubbed SplitVision (https://github.com/J35P312/SplitVision) (S1 Appendix). In short, SplitVision searches for split reads bridging each BPJ. A consensus sequence of these reads are generated through multiple sequence alignment using ClustalW [52,53] and assembly using a greedy algorithm; maximizing the length and support of each consensus sequence. The consensus sequences are then mapped to the reference genome using BWA. The exact BPs as well as any microhomology and/or insertions at the BPJs are found based on the orientation, position and cigar string of the primary and supplementary alignments of the consensus sequences. Additionally, SplitVision searches for repeat elements and SNVs close to the BPJs (<1 kb). Repeat elements are found using the USCS repeat masker [54] and SNVs are called using SAMtools [55]. Lastly, the SNVs were filtered based on the SweFreq (SweGen Variant Frequency Dataset) [56] and gnomAD (http://gnomad.broadinstitute.org). The allele frequency threshold was set to 0, removing any previously reported SNVs, and SNVs located in regions not covered by the SweGen dataset. The quality of the remaining SNVs was assessed using the Integrative Genomics Viewer (IGV) tool [57].

### 10X Genomics Chromium WGS

10X Genomics Chromium WGS was performed on sample P00 at NGI, Stockholm, Sweden. Libraries were prepared using the 10X Chromium controller and sequenced on an Illumina Hiseq Xten platform. Data was analyzed using two separate pipelines developed by 10X Genomics: the default Long Ranger pipeline (https://support.10xgenomics.com/genome-exome/software/downloads/latest) and a custom *de novo* assembly pipeline based on the Supernova *de novo* assembler (https://support.10xgenomics.com/de-novo-assembly/software/downloads/latest). The custom *de novo* assembler pipelines included mapping of raw Supernova contigs with the bwa mem intra-contig mode, as well as extraction of split contigs using a python script (https://github.com/J35P312/Assemblatron).

### Data access

The bam files of all the sequenced samples indicating SVs are deposited in European Nucleotide Archive (ENA), (S4 Table). Patients’ CNV data are reported to ClinVar (P2046_133, P2109_123, P2109_150, P2109_151, P2109_162, P2109_188, P2109_190, P2109_302, P4855_511, P4855_512, P2109_176, P1426_301, P2109_185, P5513_206, P5513_116, P5371_204) or to DECIPHER (P72, P81, P06, P74, P00). The details of in-house developed analysis tool dubbed SplitVision is provided in S1 Appendix (https://github.com/J35P312/SplitVision).

## Supporting information

S1 Fig Circos plots of all cases. All rearrangements were classified into deletions-only group (n = 8), duplications-only group (n = 7) and deletions-and-duplications group (n = 6). The copy number changes are indicated as blue (copy number gain) or red (copy number loss), and the links show connections between chromosomal BPs.
(EPS)

S2 Fig Deletions within duplications. CMA revealed two clustered duplications flanked by normal copy-number fragments (DUP-N-DUP) in four cases (P06, P4855_511, P74, P2109_150). Rearrangements are illustrated as a Circos plots and within the Circos plots as linear plot with copy number status indicated as black (normal copy number) and blue (copy number gain). However, WGS revealed cryptic nested deletions within the duplicated fragments. Thus, the deletion inside of the duplication balanced the copy-number state and resulted in DUP-N-DUP pattern observed by CMA. Linked reads showing connections between chromosomal BPs are illustrated as dashed lines. Two solutions of the final order of the genomic fragments are given, showing whether the tandem duplication is inserted before (top solution) or after (below solution) the reference region.
(EPS)

S3 Fig Signatures of MMEJ. One of the characterized BPJs in P2109_188 has very typical signatures of MMEJ: a 14bp non-templated insertion (marked in gray) followed by a 26 bp templated insertion (chr21:45466217–45466242, (-) strand, marked in green), followed by another 12 bp non-templated insertion (marked in gray), plus 3 bp and 4bp microhomologies at the 5’- (marked in blue) and the 3’-sides (marked in yellow) of the BPJ. Microhomologies are underlined and are in bold font.
(EPS)

S4 Fig Identical breakpoint junction sequences in two unrelated 2p25.3 rearrangement carriers. The 2p25.3 rearrangement breakpoint junctions that was sequenced at nucleotide level was identical in the two carriers including a SNV in *cis*, upstream of the junction (dashed red box).
(EPS)

S5 Fig Boxplots presenting the distribution of various breakpoint characteristics of the rearrangements, calculated per group. Groups are divided into deletions only, duplications only, or deletions and duplications with A) showing the number of breakpoints, B) amount of breakpoint microhomology, and C) insertions at the breakpoint junctions.
(TIFF)

S6 Fig Scatter plot and box plots of breakpoint junction characteristics, calculated per case. A) The number of breakpoints per case, B) Box plots showing the distribution of breakpoint microhomology, and C) a boxplot of the distribution of inserted sequence at the breakpoint junctions.
(TIFF)

S1 Appendix Algorithm of the software SplitVision. (DOCX)

S1 Table Parental origin investigations in seven *de novo* cases with available parental samples. (XLSX)

S2 Table Detailed characteristics of all breakpoint junctions that were solved at the nucleotide level. (XLSX)

S3 Table MIM morbid genes affected by clustered copy number variants (CNVs) and comparison of chromosomal microarray (CMA) and whole genome sequencing (WGS) reporting. (XLSX)

S4 Table Accession numbers for whole genome sequencing data on all cases in the European Nucleotide Archive (ENA). (XLS)
