# Supplementary figures and images for: Replicative and non-replicative mechanisms in the formation of clustered CNVs are indicated by whole genome characterization
Source: PLoS Genet. 2018 Nov 12;14(11):e1007780. doi: 10.1371/journal.pgen.1007780 (PMC6258378; doi:10.1371/journal.pgen.1007780)

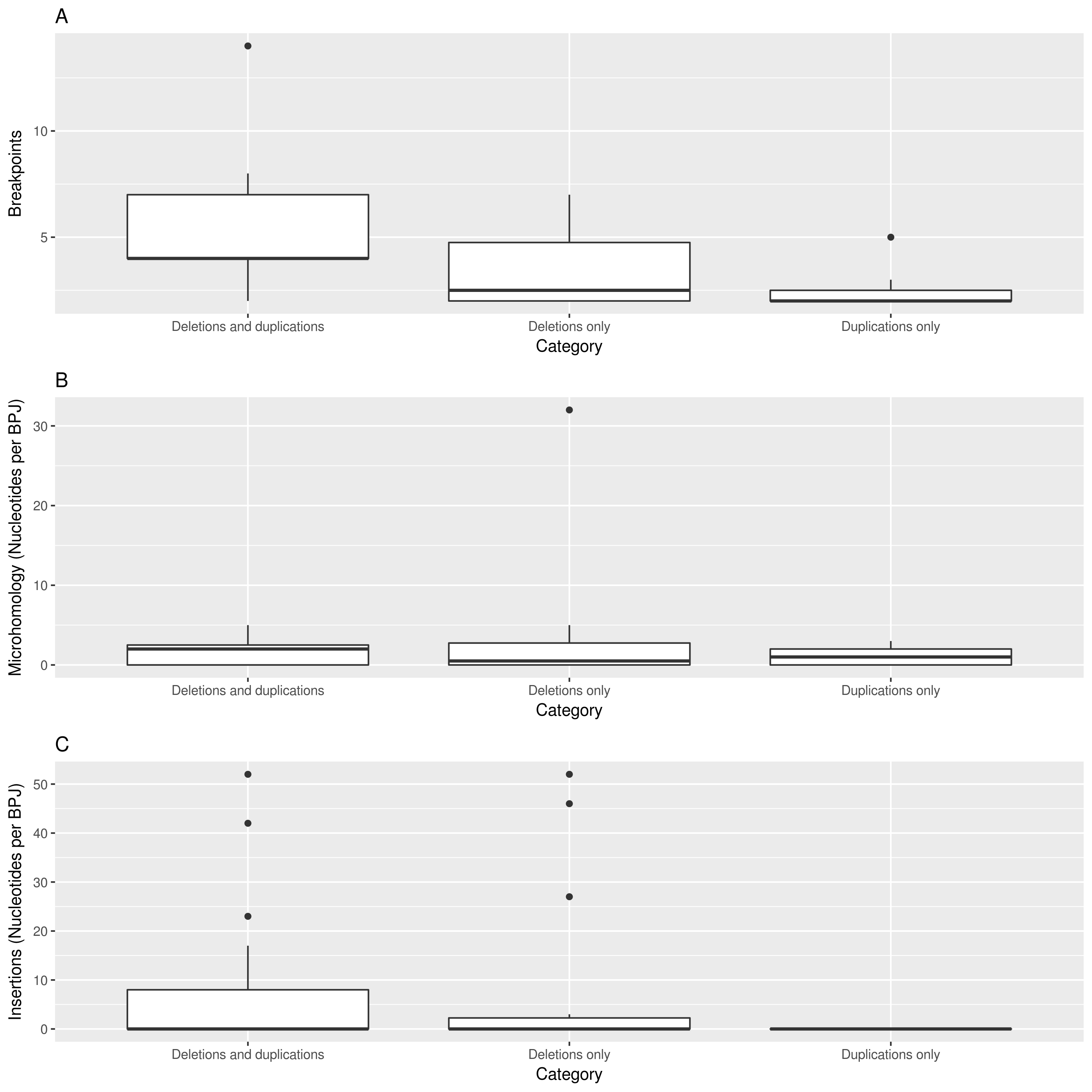

Supplement: S5 Fig — Groups are divided into deletions only, duplications only, or deletions and duplications with A) showing the number of breakpoints, B) amount of breakpoint microhomology, and C) insertions at the breakpoint junctions. (TIFF) [file pgen.1007780.s005.tiff]

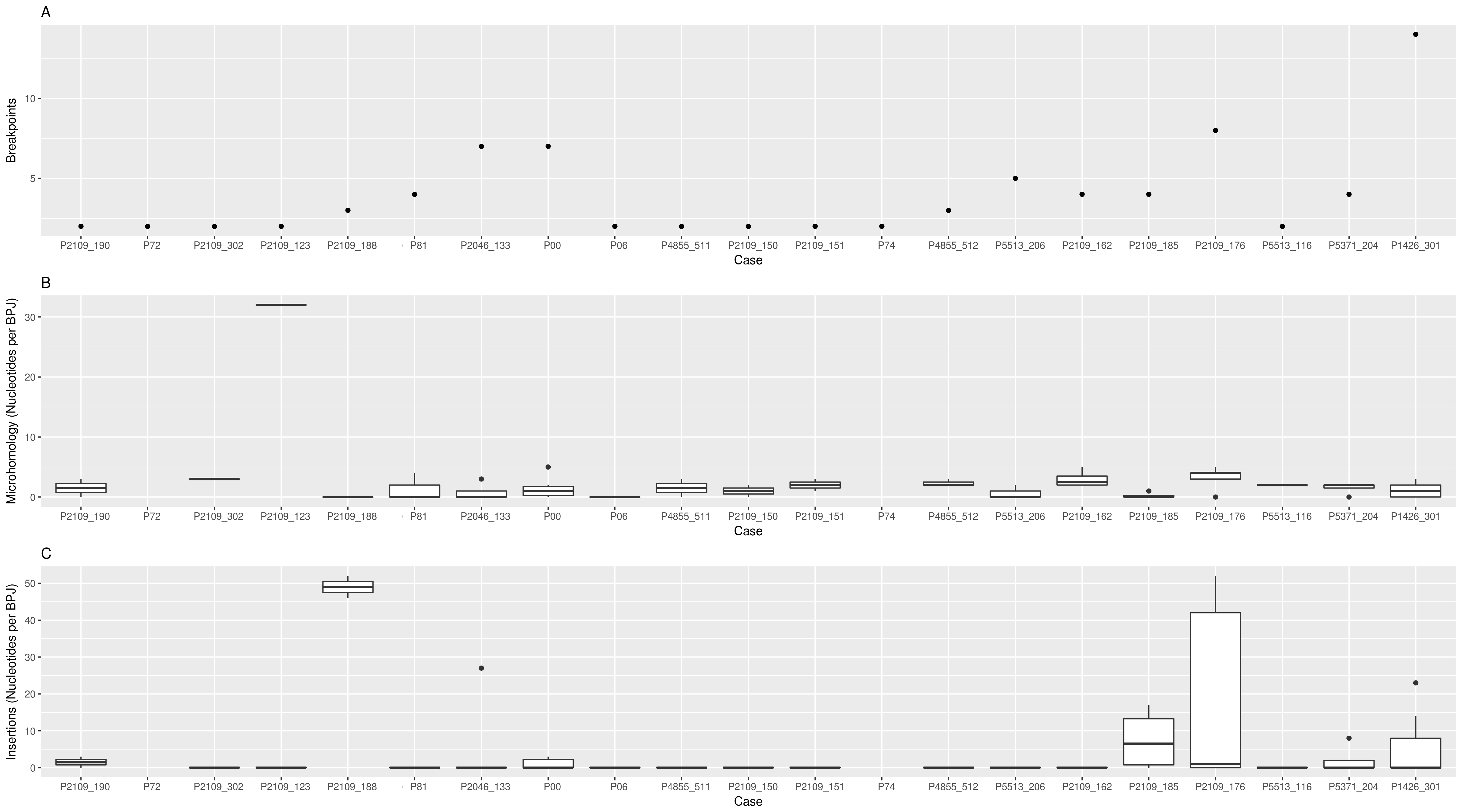

Supplement: S6 Fig — A) The number of breakpoints per case, B) Box plots showing the distribution of breakpoint microhomology, and C) a boxplot of the distribution of inserted sequence at the breakpoint junctions. (TIFF) [file pgen.1007780.s006.tiff]
