# Supplementary material for: Replicative and non-replicative mechanisms in the formation of clustered CNVs are indicated by whole genome characterization
Source: PLoS Genet. 2018 Nov 12;14(11):e1007780. doi: 10.1371/journal.pgen.1007780 (PMC6258378; doi:10.1371/journal.pgen.1007780)
Supplement: S1 Appendix — (DOCX) [file pgen.1007780.s007.docx]

**Replicative and non-replicative mechanisms in the formation of clustered CNVs are indicated by whole genome characterization**

Lusine Nazaryan-Petersen^1#^, Jesper Eisfeldt^2,3#^, Maria Pettersson^2^, Johanna Lundin^2,4^, Daniel Nilsson^2,3,4^, Josephine Wincent^2,4^, Agne Lieden^2,4^, Lovisa Lovmar^5^, Jesper Ottosson^5^, Jelena Gacic^6^, Outi Mäkitie^2,4,7,8^, Ann Nordgren^2,4^, Francesco Vezzi^9,10^, Valtteri Wirta^11,12^, Max Käller^11,12^, Tina Duelund Hjortshøj^13^, Cathrine Jespersgaard^13^, Rayan Houssari^13^, Laura Pignata^13^, Mads Bak^1^, Niels Tommerup^1^, Elisabeth Syk Lundberg^2,4^, Zeynep Tümer^13#*^, Anna Lindstrand^2,4#*^

^#^ Equal contribution

*Correspondence: [anna.lindstrand@ki.se](mailto:anna.lindstrand@ki.se), [asuman.zeynep.tuemer@regionh.dk](mailto:asuman.zeynep.tuemer@regionh.dk)

1. Wilhelm Johannsen Center for Functional Genome Research, Institute of Cellular and Molecular Medicine, University of Copenhagen, Copenhagen, 2200, Denmark

2. Department of Molecular Medicine and Surgery, Center for Molecular Medicine, Karolinska Institutet, Stockholm, 171 76 Sweden

3. Science for Life Laboratory, Karolinska Institutet Science Park, Solna, 171 21 Sweden

4. Department of Clinical Genetics, Karolinska University Hospital, Stockholm, 171 76 Sweden

5. Department of Clinical Genetics, Sahlgrenska University Hospital, 413 45 Gothenburg,

Sweden

6. Department of Clinical Genetics, Linköping University Hospital, Linköping, Sweden

7. Children’s Hospital, University of Helsinki and Helsinki University Hospital, Helsinki 00290, Finland

8. Folkhälsan Institute of Genetics, Helsinki 00290, Finland

9. SciLifeLab, Department of Biochemistry and Biophysics, Stockholm University, Stockholm, Sweden

10. Present adress: Devyser AB, Instrumentvägen 19, SE-12653 Hägersten, Sweden

11. SciLifeLab, School of Engineering Sciences in Chemistry, Biotechnology and Health, KTH Royal Institute of Technology, 100 44, Stockholm, Sweden

12.SciLifeLab, Department of Microbiology, Tumor and Cell biology, Karolinska Institutet, 171 76, Stockholm, Sweden

13. Kennedy Center, Department of Clinical Genetics, Copenhagen University Hospital, Rigshospitalet, Glostrup, 2600, Denmark

**Preprocessing**

The sequencing data was preprocessed according to the GATK best practices (ttps://software.broadinstitute.org/gatk/best-practices/bp_3step.php?case=GermShortWGS).

**FindSV analysis**

The preprocessed bam files were analysed using FindSV (https://github.com/J35P312/FindSV). FindSV was run using the default settings.

**SplitVision**

SplitVision produces summaries of breakpoint junctions (BPJ). SplitVision collects information from split reads (SR) spanning BPJ. The BPJ are provided by the user via bed or vcf files.

**Run**

SplitVision is a command line tool. SplitVision was run using the following command:

python splitvision.py --analyse --bam $1 --bed $2 --fa /home/jesperei/human_g1k_v37.fasta --repeatmask repeats.db --padding 1000 --snp_distance 1000

**Download and Install**

SplitVision is available on github:

https://github.com/J35P312/SplitVision

SplitVision is implemented in Python 2.7. And may be installed using the SplitVision install script.

**Algorithm**

SplitVision accepts a bedpe or vcf file containing BPJs of interest. Each BPJ is analysed separately. Initially SplitVision searches for SRs spanning the BPJ.

If SplitVision detects any SRs, these reads are clustered using CD-hit (1). The reads belonging to the largest cluster are selected, and a consensus sequence is computed using ClustalW2 (2). The consensus sequence is later aligned to the reference genome using BWA mem (3) (Figure 1).

If no SR were detected, local assembly is performed using ABYSS (4). If ABYSS succeeds in assembling a contig spanning the BPJ, that contig will be used as a consensus sequence.

If a consensus sequence was generated, SplitVision will use the Cigar column of the bam file to compute the exact breakpoint position. Additionally, SplitVision will search for microhomologies contained within the consensus sequence, as well as insertions.

Lastly, SplitVision will search for adjacent repeat elements and SNVs. These properties are searched for even if a consensus sequence was not found. The SNV calling is performed using Freebayes (5), any tab file could be provided as a repeat database, including the USCS repeatmasker (6).

Once completed, the statistics are printed to an excel file (Figure 1).


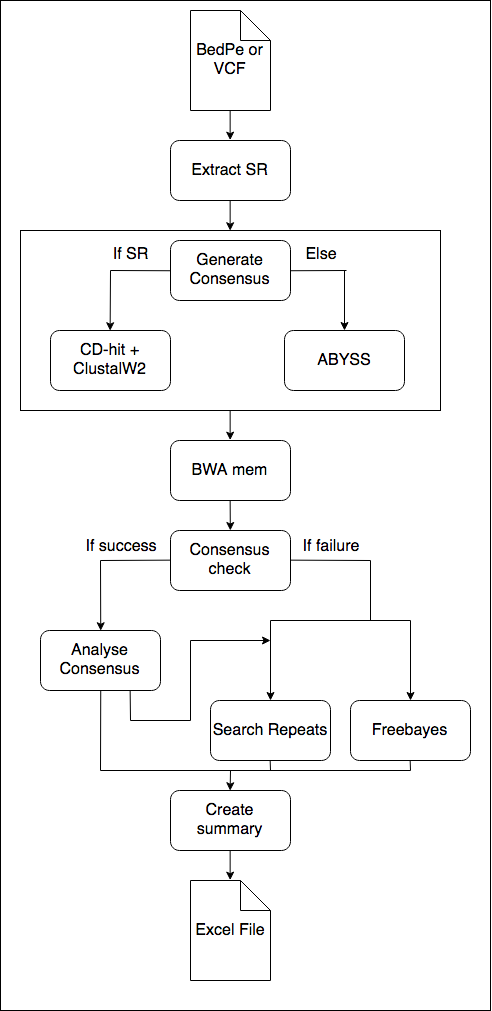


Figure 1. A schematic of the SplitVision algorithm.

**References**

1. Fu L, Niu B, Zhu Z, Wu S, Li W. CD-HIT: accelerated for clustering the next generation sequencing data. Bioinformatics [Internet]. 2012;28(23):3150–2. Available from: http://www.ncbi.nlm.nih.gov/pubmed/23060610

2. Larkin M a, Blackshields G, Brown NP. ClustalW2 and ClustalX version 2. … [Internet]. 2007;1–2. Available from: http://bioinformatics.oxfordjournals.org/content/23/21/2947.short

3. Li H. Aligning sequence reads, clone sequences and assembly contigs with BWA-MEM. arXiv Prepr arXiv [Internet]. 2013;0(0):3. Available from: http://arxiv.org/abs/1303.3997

4. Simpson JT, Wong K, Jackman SD, Schein JE, Jones SJM, Birol I. ABySS: A parallel assembler for short read sequence data. Genome Res. 2009;19(6):1117–23.

5. Garrison E, Marth G. Haplotype-based variant detection from short-read sequencing. 2012;1–9. Available from: http://arxiv.org/abs/1207.3907

6. Tarailo-Graovac M, Chen N. Using RepeatMasker to identify repetitive elements in genomic sequences. Current Protocols in Bioinformatics. 2009.
